# Characterization of Mild and Moderate Dysarthria in Parkinson’s Disease: Behavioral Measures and Neural Correlates

**DOI:** 10.3389/fnagi.2022.870998

**Published:** 2022-05-16

**Authors:** Hanna Steurer, Ellika Schalling, Erika Franzén, Franziska Albrecht

**Affiliations:** ^1^Department of Clinical Science, Intervention and Technology (CLINTEC), Division of Speech and Language Pathology, Karolinska Institutet, Stockholm, Sweden; ^2^R&D Unit, Stockholms Sjukhem, Stockholm, Sweden; ^3^Department of Public Health and Caring Sciences, Speech-Language Pathology, Uppsala University, Uppsala, Sweden; ^4^Department of Neurobiology, Care Sciences and Society, Division of Physiotherapy, Karolinska Institutet, Karolinska University Hospital, Women’s Health and Allied Health Professionals, Stockholm, Sweden

**Keywords:** dysarthria, magnetic resonance imaging, Parkinson’s disease, superior temporal gyrus (STG), speech, language, cognition

## Abstract

**Purpose:**

Alterations in speech and voice are among the most common symptoms in Parkinson’s disease (PD), often resulting in motor speech disorders such as hypokinetic dysarthria. We investigated dysarthria, verbal fluency, executive functions, and global cognitive function in relation to structural and resting-state brain changes in people with PD.

**Methods:**

Participants with mild-moderate PD (*n* = 83) were recruited within a randomized controlled trial and divided into groups with varying degrees of dysarthria: no dysarthria (noDPD), mild dysarthria (mildDPD), moderate dysarthria (modDPD), and also combined mildDPD and modDPD into one group (totDPD). Voice sound level and dysphonia, verbal fluency, motor symptoms, executive functions, disease severity, global cognition, and neuroimaging were compared between groups. Gray matter volume and intensity of spontaneous brain activity were analyzed. Additionally, regressions between behavioral and neuroimaging data were performed.

**Results:**

The groups differed significantly in mean voice sound level, dysphonia, and motor symptom severity. Comparing different severity levels of dysarthria to noDPD, groups differed focally in resting-state activity, but not in brain structure. In totDPD, lower scores on semantic verbal fluency, a composite score of executive functions, and global cognition correlated with lower superior temporal gyrus volume.

**Conclusion:**

This study shows that severity of dysarthria may be related to underlying structural and resting-state brain alterations in PD as well as behavioral changes. Further, the superior temporal gyrus may play an important role in executive functions, language, and global cognition in people with PD and dysarthria.

## Introduction

Parkinson’s disease (PD) is one of the most common neurodegenerative progressive diseases. Prevalence increases with age up to 1,903/100 000 in people 80 years and older (Pringsheim et al., 2014). Up to 90% of people with PD report changes in speech, voice, and communication (Logemann et al., 1978; Hartelius and Svensson, 1994; Schalling et al., 2017). For many with PD, speech changes result in hypokinetic dysarthria, a motor speech disorder. Common symptoms are hypophonia or reduced vocal loudness, dysphonia, reduced articulatory precision, and changes of speech rate (Darley et al., 1969). Early in the disease progression (Logemann et al., 1978; Ho et al., 1998; Rusz et al., 2011) the most common symptoms are voice disorders such as reduced voice sound level and impaired voice quality including dysphonia. In fact, vocal dysfunction may be one of the earliest signs of motor impairment in PD and thus a potential prognostic biomarker in PD (Ma et al., 2020). Changes in the perception of the loudness of the own voice have also been shown in people with PD with difficulties scaling up and regulating effort required to speak with normal intensity (Ho et al., 2000). Further common in people with PD are changes related to language and cognition, e.g., word-finding difficulties or getting off-topic when speaking (Miller et al., 2006; Schalling et al., 2017). Studies have shown that communicative impairment as well as cognitive and psychiatric changes in PD result in reduced participation in various activities and reduced quality of life (Yorkston et al., 2017; Dashtipour et al., 2018).

Changes in brain activity or structure related to speech impairment in PD are still far from understood. A few studies using resting-state magnetic resonance imaging (rsMRI) or functional MRI (fMRI) have identified diverse connectivity changes in relation to speech in people with mild-moderate PD (Arnold et al., 2014; Elfmarková et al., 2016; Brabenec et al., 2017; Baumann et al., 2018). Morphological brain changes underlying hypokinetic dysarthria were investigated in a recent study of 134 participants with PD (Chen et al., 2020). Atrophy in the right precentral cortex and the right fusiform gyrus were found to be associated with hypokinetic dysarthria. Of note, however, the diagnosis of hypokinetic dysarthria was based on a self-report instrument mainly used to assess psychosocial consequences of voice disorders (The Voice Handicap Index) (Jacobson et al., 1997).

Approaching identification of the brain regions that in typical speakers underly speech, a voxel-based meta-analysis of healthy controls singing and reading during fMRI found following regions to be activated: frontal and Rolandic operculum, larynx motor cortex, supplementary motor area, cingulate motor area, superior temporal gyrus, primary auditory cortex, putamen, thalamus, cerebellum (Lobule VI, Vermis V/VI) (Brown et al., 2009). These regions have been identified as the common convergence of 11 studies and build the basis for the regions-of-interest (ROI) selection in the present study.

This study aimed to better understand mechanisms behind dysarthria in people with mild-moderate PD by linking characterization of speech and voice impairment (measured acoustically, perceptually, and by self-rating) to structural and resting-state brain changes (measured by MRI) as well as to linguistic (verbal fluency) and cognitive factors (executive functions and screening of cognitive functions). Participants were stratified into groups with different severity levels of dysarthria (no/mild/moderate) based on perceptual analysis. Hypothesis-driven ROI analyses to investigate structural and functional changes, as well as whole-brain correlations to explore related regions, were performed and compared between groups. ROIs were based on the meta-analysis of speech introduced above (Brown et al., 2009), but since literature shows that neural correlates of speech may not be solely right-lateralized, we selected ROIs bilaterally. Furthermore, correlations of structural and resting-state brain changes with acoustic measures of speech and voice, verbal fluency, general cognition, and executive functions were performed.

## Materials and Methods

### Participants

Participants with PD were recruited within the framework of a randomized controlled trial (RCT), the EXPANd trial [for details see study protocol (Franzén et al., 2019)]. In short, inclusion criteria were age ≥ 60 years, idiopathic PD diagnosed by neurologist, Hoehn and Yahr II-III, and a Montreal Cognitive Assessment (MoCA) score ≥ 21. Exclusion criteria were other neurodegenerative or neuromuscular diseases, having participated in a balance or speech exercise program during the last 6 months, metal implants, claustrophobia, and severe hearing impairment. For this study, we included cross-sectional data of participants of the RCT with available MRI data and speech assessment (*N* = 83). Participants with PD were assessed with a comprehensive test battery including assessment of speech, voice and communication, a neuropsychological test battery, assessment of balance and motor impairment as well as a structural (sMRI) and rsMRI acquisition. During baseline assessments all participants of the EXPANd trial were asked to report their medication intake. Participants were assessed in the ON stage of PD medication and asked to ensure that no changes in medication were made during the study period. One participant reported medicating with psychoanaleptics.

### Assessment of Speech, Voice, and Communication

#### Speech Recordings

Speech recordings were performed according to standardized routines for high-quality recordings in a sound-proof recording studio with the equipment Sony Digital Audio Tape Deck DTC-ZE700 in a recording studio in a university department. Sopran (version 1.0.22© Tolvan Data), a software for sound processing and analyses, and a head microphone (Sennheiser HSP 4 with an MZA 900 P phantom power adapter) which was calibrated for a pre-determined mouth-microphone distance of 15 cm were used for all recordings. A recording protocol including different speech tasks was applied, providing a basis for acoustic and auditory-perceptual analyses; two approaches used for assessing voice function and quality.

#### Voice Sound Level

Acoustic analysis of voice sound level in reading “Trapetskonstnären,” a Swedish standardized phonetically balanced text constructed for evaluation of neuromotor speech disorders (Hartelius, 2015), was performed on the studio recordings. The software Sopran (version 1.0.22© Tolvan Data) was used for analysis. To reduce the impact of low-frequency background noise on the sound level, a C-weighted decibel (dBC) was used to report the voice sound level.

#### Acoustic Voice Quality Index

The AVQI is a composite measure that combines several acoustic parameters to obtain a single score for the estimation of dysphonia. The equation of the AVQI includes the smoothed cepstral peak prominence (CPPS), harmonics-to-noise ratio (HNR), shimmer local (SL), shimmer local dB (SLdB), general slope of the spectrum (Slope), and tilt of the regression line through the spectrum (Tilt). The parameters are weighted together through linear regression analysis and converted to a score on a linear scale between 0 and 10. The limit for what is considered a dysphonic voice according to AVQI varies across languages. For this study, the limit was set to 2.95 (validated for Dutch speakers). Scores below the limit value are considered to represent a normal voice function (Maryn et al., 2009). AVQI analyses were performed using extractions from the speech recordings of each participant. First, the middle 3 s were extracted from a sustained vowel [a:], with a margin of 0.10 s. In the recordings which included repeated attempts at the sustained vowel, the last attempt was consistently used for analysis. Second, extractions of a pre-chosen 45 syllables of reading the text “Trapetskonstnären” were used.

#### Perceptual Listener Ratings for Assessment of Dysarthria

Evaluation of speech and voice function is commonly based on clinical tests and/or speech recordings using test protocols. These include speech and voice tasks such as sustained phonation, syllable repetition, reading of words, sentences, and a short paragraph as well as spontaneous speech (Duffy, 2016; Brabenec et al., 2017). However, there is no consensus-based gold standard for the evaluation of speech and voice function. Notably, previous studies investigating neural correlates of speech differ in the definition of speech impairment. Most neuroimaging studies use PD-specific rating scales and questionnaires, such as the MDS-UPDRS III (speech item 3.1), to define the speech impairment (New et al., 2015; Brabenec et al., 2017; Manes et al., 2018). These are clinical screening tools for motor impairment including two items related to speech, but an insufficiently detailed measure of dysarthria in PD and are typically not administered by speech-language pathologists. In contrast, the study of Baumann et al. (2018) relied on the expert assessment by a movement disorder specialist and a speech-language pathologist to assess dysarthria in PD. Hence, further research in the development of such approaches is needed to pave the way for better comparability between studies.

The test battery of the EXPANd trial included the Dysarthria Assessment (Hartelius, 2015); a Swedish standardized clinical test used for assessing dysarthria. The test includes assessment of respiration, phonation, oro-motor and velopharyngeal function, prosody, and intelligibility. Each domain consists of several tasks with are rated on a scale from 0 to 3 (0 = no deviation, 1 = mild deviation, 2 = moderate deviation, and 3 = marked deviation). The scores for each domain are summed up and divided by the number of tasks in each domain to yield an average score. Subsequently, the total score of the dysarthria test is calculated by taking the average of the average scores from all domains. The total score is considered to reflect the severity stage of dysarthria (i.e., no/mild/moderate/severe). However, the severity of dysarthria was mild in this cohort of PD participants and the Dysarthria Assessment did not sufficiently reflect the clinically observed dysarthria in a reliable way (participants presented with generally very low scores, data not shown). Consequently, we attempted to elucidate the participants’ type and degree of speech and voice impairment using perceptual listener ratings. These were performed in consensus by two clinicians with several years of experience of neuromotor speech disorders from clinical work and research. Speech recordings of participants reading the first 142 syllables of a Swedish text developed for evaluation of motor speech disorders [(Hartelius, 2015) “Trapetskonstnären”] were used. Inspired by a general protocol for perceptual listener ratings (Wannberg et al., 2016), impression of degree of overall speech impairment and impairment in three general areas of speech production (articulation, voice function, and prosody) were rated as 0 = no deviation, 1 = mild deviation, or 2 = marked deviation. Based on the average scores from the four ratings, participants were divided into three groups with varying degrees of dysarthria in PD; 0–0.25: no dysarthria in PD (noDPD), 0.5–1.25: mild dysarthria (mildDPD), 1.5–2.0: moderate dysarthria (modDPD). For some analyses, mildDPD and modDPD were combined into one group representing mild-moderate dysarthria in PD (totDPD).

To enable analysis of intra-rater reliability, the participants were numbered and 25% (*n* = 22) of the numbers were randomly drawn, and the corresponding sound files were added twice to the list of sound files used for rating. For these duplicates, the second rating was consistently used for grouping the participant into noDPD/mildDPD/modDPD. To validate the perceptual rating of the parameter voice function used for assessment of dysarthria, multiple linear regression analysis was performed.

#### Composite Score Self-Rated Dysarthria

To reflect two perspectives of dysarthria, self-rated dysarthria was used a*s* a complement to the expert perceptual listener ratings. The test battery included the Swedish standardized version of the Questionnaire of Acquired Speech Disorders (QASD) (in Swedish: Självsvarsformulär om Förvärvade Talsvårigheter, SOFT) including 30 items addressing speech function, speech activity, and communicative participation (Hartelius, 2015). To reduce the number of variables, we computed a composite score for self-rated dysarthria. For the self-rated dysarthria score, the following QASD items were chosen: 1 (“my speech is slow”), 2 (“my speech is unclear”), 3 (“some sounds or letters are difficult for me to say”), and 4 (“I often sound hoarse”). These items were selected on the basis that they cover the different aspects of speech production best reflecting the protocol for perceptual listener ratings. All scores of the tests were standardized into z-scores by the mean and standard deviation. We compared several models for the construction of the self-rated dysarthria score. First the maximum likelihood estimation and second the robust diagonally weighted least square estimation using a polychoric model which is suitable for categorical data. The robust diagonally weighted least square estimation was chosen since it provided the best fit (robust RMSEA = 0.079, robust Comparative Fit Index = 0.981, robust Tucker-Lewis Index = 0.944). The included items had the following factor loadings: QASD 1 = 1.000, QASD 2 = 1.504, QASD 3 = 0.938, QASD 4 = 0.561. Since QASD item 4 showed a low factor loading, it was not included in the final composite score. Sum scores for each participant were computed by multiplying the z-scores of the tests with their factor loadings and adding them together to derive final composite scores.

### Assessment of Motor Impairment

The participants’ disease stage and severity were assessed using the Movement Disorder Society Unified Parkinson’s Disease Rating Scale (MDS-UPDRS) (Goetz et al., 2008). Balance performance was assessed with the Mini-BESTest (Franchignoni et al., 2010). The last item on the Mini-BESTest [Timed Up and Go (TUG) and TUG cognitive (TUGcog)] was also used as separate variables of functional mobility. In the TUG standing up from a chair, walking 3 m, turning around, returning to the chair, and sitting down is assessed as the number of seconds to complete the movement sequence. A serial subtraction task was used in TUGcog and the difference in seconds between TUG and TUGcog (TUGcog − TUG = TUGdiff), respectively, were utilized as outcomes. Gait speed was collected using an electronic walkway system (GAITRite ^®^, active zone: 8.3 m, CIR Systems, Inc., Havertown, PA, United States). At a self-selected speed, participants walked six times back and forth on the walkway. Participants started walking 3 m before and stopped 3 m after the end of the walkway, which accounts for acceleration and deceleration phases.

### Neuropsychological Tests

Within the EXPANd trial, participants were assessed with a large neuropsychological test battery comprising the domains executive function, attention/working memory, episodic memory, and visuospatial functions (Franzén et al., 2019). This test battery enabled the classification of mild cognitive impairment, previously described by Johansson and colleagues (Johansson et al., 2021). In this study we focused on the following scales to assess cognition and executive function: the Delis-Kaplan Executive Function System (D-KEFS) (Fine and Delis, 2011), the Wechsler Adult Intelligence Scale-Fourth Edition (WAIS-IV) (Wechsler, 1955), and MoCA (Borland et al., 2017).

#### Composite Score Executive Functions

A composite score for executive functions was created by merging four tests: (1) letter fluency, (2) the verbal fluency test (category switching), (3) the color-word interference test (switch condition, 1–3 from D-KEFS), and (4) the digit span total score from WAIS-IV. All scores were standardized into z-scores by the mean and standard deviation. Four models were compared: with and without the color-word interference test and applying either the maximum likelihood estimation or the robust diagonally weighted least square estimation. We tested with and without the color-word interference test due to its skewed distribution. The model including all four tests based on the robust diagonally weighted least square estimation yielded the best fit values (robust RMSEA = 0.044, robust Comparative Fit Index = 0.995, robust Tucker-Lewis Index = 0.984). Factor loadings of the model were as following: verbal fluency = 1.000, the verbal fluency test (category switching) = 0.887, the color-word interference test (switch condition) = −0.855, the digit span total score = 0.813. For the final composite scores, sum scores for each participant were computed by multiplying the z-scores of the tests with their factor loadings and adding them together.

### Magnetic Resonance Imaging

Structural MRI data were acquired on a 3T Phillips Ingenia scanner using a T1-weighted sequence with the following parameters: repetition/echo time (TR/TE) = 6.1/2.8 ms and voxel-size of 1 × 1 × 1 mm. rsMRI was acquired with an echo-planar imaging (EPI) sequence with TR/TE = 2073/7.3 ms, 224 volumes, 40 slices, 75° flip angle, a voxel-size of 3.5 × 3.5 × 3.5 mm and eyes open looking at a fixation cube.

#### Voxel-Based Morphometry

Structural magnetic resonance imaging data were preprocessed by the standard pipeline of CAT12 (version 12.7) (Gaser and Dahnke, 2016). MRI data were controlled for quality by “weighted overall image quality” and measures of “sample homogeneity” in CAT12 (Gaser and Dahnke, 2016). No participants had to be excluded. We ran voxel-based morphometry analyses where first hypothesis-driven ROIs were tested and compared between groups and second exploratory whole-brain comparisons. ROI masks were created and analyzed with the WFU PickAtlas toolbox (version 3.0.5) (Maldjian et al., 2003) in SPM12 (version 7771). ROIs were based on an fMRI meta-analysis of speech and singing in healthy controls (Brown et al., 2009). Areas include the frontal and Rolandic operculum, larynx motor cortex, supplementary motor area, cingulate motor area, superior temporal gyrus, primary auditory cortex, putamen, thalamus, and cerebellum (Lobule VI, Vermis V/VI). We used separate masks for the left and right hemispheres. Furthermore, voxel-wise whole-brain regression analyses were calculated on the following variables of interests in separate models in SPM12: MoCA, MDS-UPDRS total, MDS-UPDRS III, executive functions composite score, self-rated dysarthria composite score, D-KEFS verbal fluency semantic score, D-KEFS verbal fluency FAS score, AVQI, and voice sound level.

#### Resting-State Magnetic Resonance Imaging

The images were preprocessed through the HiveDB (Muehlboeck et al., 2014) using the SPM12 standard pipeline for rsMRI and analyzed with DPARSF (Chao-Gan and Yu-Feng, 2010) (DPABI version 6.0). In short, images were reoriented, slice time corrected, realigned, co-registered with their T1 images, normalized, and smoothed (full width at half-maximum, FWHM = 3 mm^3^). Framewise displacement (FD) mean and maximum values were calculated to assess motion (Matlab R2019b) (Power et al., 2014). Participants with mean FD > 2 mm were excluded. This was the case for one participant with mildDPD. None of the groups of PD with and without dysarthria (noDPD, mildDPD, modDPD, totDPD) differed in their mean FD values. Each participant’s co-registered white matter (WM) and cerebrospinal fluid (CSF) segmented images were binarized with a threshold of 0.3 in FSL (version 6.0.3) and used as a mask to obtain nuisance regressors. To obtain 5 WM and 5 CSF regressors, we applied the aCompCor approach using the function of Mascali and colleagues (Mascali et al., 2021). The Friston 24-model (Friston et al., 1996) was chosen for motion regression: 6 rigid body motion parameters, their squares, their temporal derivatives, and the squares of the temporal derivatives (Nichols, 2012). A linear model was constructed to regress out nuisance (Mascali et al., 2021). A measure of the intensity of regional spontaneous brain activity, fraction of amplitude of low frequency fluctuations (fALFF) (Chao-Gan and Yu-Feng, 2010) was computed on the nuisance regressed, smoothed data but without filtering. It is a voxel-wise measure of the full frequency range. We analyzed the z-standardized maps where every voxel is divided by the mean fALFF within the whole-brain [(fALFF values-mean)/standard deviation]. We further analyzed degree centrality and regional homogeneity which we report in the Supplementary Material. We performed the same ROI, whole-brain, and regression analyses as mentioned in the sMRI analyses.

### Statistical Analyses

Comparisons were analyzed for behavioral as well as brain measures between: (1) moderate and no dysarthria (modDPD vs. noDPD), (2) mild and no dysarthria (mildDPD vs. noDPD), (3) mild and moderate dysarthria (mildDPD vs. modDPD), and (4) dysarthria and no dysarthria (totDPD vs. noDPD). Brain-behavior correlations were only obtained for the comparisons of the groups with and without dysarthria (totDPD vs. noDPD).

To test for differences in demographic, speech and voice, clinical, motor, and cognitive variables between the PD-dysarthria groups, Kruskal Wallis tests were performed in RStudio (Version 1.3.1073) (RStudio Team, 2015).

Missing values were replaced by the group mean. We imputed values for three variables (voice sound level for two participants and executive functions composite score for one participant). If there was more than one missing data point for a certain participant, the participant was excluded from analyses. Four participants were missing QASD data and hence were excluded from those analyses.

### Deviations From Preregistration

The hypotheses, methods, and analyses for this project were preregistered at aspredicted.org and is available at the EXPANd RCT OSF page.^1^ We deviated from this protocol and added analyses. We additionally analyzed comparisons using AVQI. Analyses of rsMRI data were not preregistered and performed after analyzing sMRI data. Furthermore, we preregistered to use mild cognitive impairment (MCI) classification for regression analyses. However, we refrained from using the MCI classification since this was a dichotomous scale which decreases power and yields less differentiated information about cognitive variability than MoCA and our composite score for executive functions.

## Results

### Clinical Assessment of Dysarthria

Our cohort was stratified into 44 mildDPD, 20 modDPD, and 19 noDPD (Tables 1, 2). The absolute intra-rater agreement of the dysarthria grouping was 86.3%. For 13 participants the first and second ratings were identical. For six participants the ratings differed but did not affect the grouping. For three participants the ratings differed and affected the grouping.

**TABLE 1:** Demographic, motor, cognitive, and clinical data for participants with PD stratified into dysarthria severity levels and PD without dysarthria.

	PD moderate dysarthria (*N* = 20)	PD mild dysarthria (*N* = 44)	PD no dysarthria (*N* = 19)	Total (*N* = 83)	*p*-value
Age, years					0.7691^1^
Mean (CI)	70.95 (68.17, 73.73)	71.02 (69.19, 72.85)	70.00 (67.16, 72.84)	70.77 (69.48, 72.06)	
Sex					0.1272^2^
Male	13 (65.0%)	23 (52.3%)	15 (78.9%)	51 (61.4%)	
Female	7 (35.0%)	21 (47.7%)	4 (21.1%)	32 (38.6%)	
Disease duration, years					0.2061^1^
Mean (CI)	5.15 (2.69, 7.61)	5.93 (4.54, 7.32)	3.37 (2.20, 4.54)	5.16 (4.19, 6.13)	
Education, years					0.3251^1^
Mean (CI)	15.16 (13.73, 16.59)	14.56 (13.56, 15.55)	15.74 (14.37, 17.11)	14.98 (14.29, 15.66)	
Hoehn and Yahr					0.329^1^
Mean (CI)	2.35 (2.12, 2.58)	2.18 (2.06, 2.30)	2.21 (2.01, 2.41)	2.23 (2.14, 2.32)	
LEDD, mg					0.681^1^
Mean (CI)	571.80 (431.77, 711.83)	564.99 (457.33, 672.64)	463.47 (319.19, 607.75)	543.39 (471.60, 615.18)	
MDS-UPDRS I					0.9441^1^
Mean (CI)	8.75 (5.89, 11.61)	8.93 (7.25, 10.61)	8.26 (5.96, 10.56)	8.73 (7.54, 9.93)	
MDS-UPDRS II					0.2351^1^
Mean (CI)	11.60 (8.81, 14.39)	9.77 (8.07, 11.48)	8.74 (6.00, 11.47)	9.98 (8.72, 11.23)	
MDS-UPDRS III					0.0361^1^
Mean (CI)	36.45 (31.16, 41.74)	28.84 (26.15, 31.53)	30.58 (23.97, 37.19)	31.07 (28.66, 33.48)	
MDS-UPDRS IV					0.9691^1^
Mean (CI)	0.55 (−0.29, 1.39)	0.91 (0.16, 1.65)	0.68 (−0.12, 1.49)	0.77 (0.31, 1.24)	
MDS-UPDRS Total					0.1891^1^
Mean (CI)	57.35 (47.37, 67.33)	48.45 (43.99, 52.92)	48.00 (38.72, 57.28)	50.49 (46.62, 54.37)	
PDQ-39					0.8581^1^
Mean (CI)	22.02 (15.98, 28.06)	20.42 (16.78, 24.05)	19.74 (13.51, 25.97)	20.65 (17.97, 23.33)	
Executive function					0.3261^1^
Mean (CI)	−0.40 (−1.65, 0.84)	−0.03 (−0.75, 0.69)	0.70 (−0.40, 1.81)	0.05 (−0.48, 0.58)	
MoCA					0.7651^1^
Mean (CI)	25.50 (24.18, 26.82)	26.09 (25.37, 26.81)	26.11 (25.08, 27.13)	25.95 (25.42, 26.48)	
Verbal fluency semantic score					0.0651^1^
Mean (CI)	35.50 (30.66, 40.34)	38.55 (35.71, 41.38)	43.79 (37.98, 49.60)	39.01 (36.71, 41.31)	
Verbal fluency FAS					0.3761^1^
Mean (CI)	42.95 (36.51, 49.39)	41.98 (37.67, 46.28)	46.42 (39.61, 53.23)	43.23 (40.17, 46.29)	
Voice sound level, dB					***0.0161*** ^1^
Mean (CI)	68.69 (66.94, 70.43)	70.36 (69.38, 71.34)	72.43 (70.15, 74.72)	70.41 (69.56, 71.26)	
QASD total					0.2101^1^
Mean (CI)	0.67 (0.45, 0.90)	0.57 (0.44, 0.70)	0.43 (0.24, 0.62)	0.56 (0.47, 0.66)	
QASD self-assessed speech impairment					0.9131^1^
Mean (CI)	0.15 (−0.52, 0.82)	−0.03 (−0.40, 0.34)	−0.00 (−0.75, 0.75)	0.02 (−0.27, 0.31)	
QASD word finding difficulty					0.8501^1^
Mean (CI)	0.79 (0.49, 1.09)	0.93 (0.66, 1.21)	0.89 (0.65, 1.12)	0.89 (0.72, 1.06)	
AVQI					***0.0351*** ^1^
Mean (CI)	4.40 (4.09, 4.70)	3.91 (3.65, 4.18)	4.00 (3.56, 4.44)	4.05 (3.86, 4.23)	

*^1^Kruskal-Wallis rank sum test, ^2^Pearson’s Chi-squared test. p-values > significance level 0.05 in bold. AVQI, Acoustic Voice Quality Index, higher scores reflect a higher level of dysphonia; CI, Confidence Interval; Executive function, composite score with higher scores representing better performance; Hoehn and Yahr, higher levels represent higher disease stage; LEDD, levodopa equivalent daily dose, higher scores represent higher medication intake; MDS-UPDRS, The Movement Disorder Society-sponsored Revision of the Unified Parkinson’s Disease Rating Scale, higher scores reflect more Parkinson’s disease-related symptoms; MoCA, Montreal Cognitive Assessment, ≥26 are considered to indicate no cognitive impairment; PDQ-39, 39 item PD Questionnaire, higher scores reflect a higher PD-specific health related quality; QASD, Questionnaire on Acquired Speech Disorders, scores represent the mean score of rated items on a 4-point scale from 0 to 3 (0 = definitely false, 1 = mostly false, 2 = mostly true, and 3 = definitely true); Verbal fluency FAS/semantic, higher scores represent a better performance; Voice sound level dB, voice sound level reported in C-weighted decibel with higher scores representing higher voice sound level.*

**TABLE 2:** Variables used for neuroimaging comparisons stratified into PD with and without dysarthria.

	PD mild and moderate dysarthria (*N* = 64)	PD no dysarthria (*N* = 19)	Total (*N* = 83)	*p*-value
Age, years				0.470^1^
Mean (CI)	71.00 (69.51, 72.49)	70.00 (67.16, 72.84)	70.77 (69.48, 72.06)	
Sex				0.074^2^
Male	36 (56.2%)	15 (78.9%)	51 (61.4%)	
Female	28 (43.8%)	4 (21.1%)	32 (38.6%)	
Disease duration, years				0.098^1^
Mean (CI)	5.69 (4.50, 6.88)	3.37 (2.20, 4.54)	5.16 (4.19, 6.13)	
MDS-UPDRS III				0.488^1^
Mean (CI)	31.22 (28.66, 33.78)	30.58 (23.97, 37.19)	31.07 (28.66, 33.48)	
Executive function				0.148^1^
Mean (CI)	−0.15 (−0.76, 0.46)	0.70 (−0.40, 1.81)	0.05 (−0.48, 0.58)	
MoCA				0.857^1^
Mean (CI)	25.91 (25.28, 26.53)	26.11 (25.08, 27.13)	25.95 (25.42, 26.48)	
Verbal fluency semantic score				***0.031*** ^1^
Mean (CI)	37.59 (35.17, 40.01)	43.79 (37.98, 49.60)	39.01 (36.71, 41.31)	
Verbal fluency FAS				0.168^1^
Mean (CI)	42.28 (38.80, 45.76)	46.42 (39.61, 53.23)	43.23 (40.17, 46.29)	
Voice sound level, dB				***0.024*** ^1^
Mean (CI)	69.83 (68.96, 70.69)	72.43 (70.15, 74.72)	70.41 (69.56, 71.26)	
AVQI				0.526^1^
Mean (CI)	4.06 (3.86, 4.27)	4.00 (3.56, 4.44)	4.05 (3.86, 4.23)	

*^1^Kruskal-Wallis rank sum test, ^2^Pearson’s Chi-squared test. p-values > significance level 0.05 in bold. AVQI, Acoustic Voice Quality Index, higher scores reflect a higher level of dysphonia; CI, Confidence interval; Executive function, composite score with higher scores representing better performance; MDS-UPDRS, The Movement Disorder Society-sponsored Revision of the Unified Parkinson’s Disease Rating Scale, higher scores reflect more Parkinson’s disease-related symptoms; MoCA, Montreal cognitive assessment, ≥26 are considered to indicate no cognitive impairment; Voice sound level dB, voice sound level reported in C-weighted decibel with higher scores representing higher voice sound level.*

We also calculated intra-rater reliability regarding how *consistent* the raters were in their total score using a two-way random intraclass-correlation (ICC) [2,1 *consistency*] (Weir, 2005). The average measure ICC was 0.87 with a 95% confidence interval from 0.783 to 0.865 [*F*_(162,972)_ = 5.775, *p* < 0.001]. According to Koo and Li (2016), this corresponds to good reliability.

Multiple linear regression analysis was performed to validate the perceptual rating of the parameter voice function. The predictor variables voice sound level and degree of dysphonia (AVQI) were entered into the regression model. Voice sound level was a significant predictor for voice function [*F*_(6_,_251)_ = −0.051, *p* = 0.005, *R*^2^_adj_ = 0.116] (Supplementary Table 1). AVQI was not a significant predictor of voice function.

### Demographic, Speech, Voice, Motor, and Cognitive Variables

Kruskal-Wallis tests showed no difference in demographic and basic clinical measures. Between the three groups, statistically significant differences were found in voice sound level, degree of dysphonia (AVQI), and motor symptom severity (MDS-UPDRS-III) (Table 1). Measures of verbal fluency semantic score and functional mobility (TUG) were marginally significant.

Assessment of dyskinesia (MDS-UPDRS item 3.18A) showed that 10% of the whole study cohort had signs of dyskinesia (*n* = 8 out of 83) (see Supplementary Table 2 for details).

The cognitive assessment revealed that 71% of the participants in the whole cohort were described by normal cognition, whereas 29% were classified as mild cognitive impairment. Stratifying the proportion of participants with mild cognitive impairment into the different severity levels of dysarthria underlined that the presence of mild cognitive impairment is rather stable across the dysarthria severity level groups (Supplementary Table 3).

### Structural Alterations

#### Group Comparison

We found no significant statistical differences in whole-brain or ROI gray matter volume between the groups with different severity levels of dysarthria (noDPD, totDPD, modDPD).

#### Regression Analyses

**FIGURE 1:**
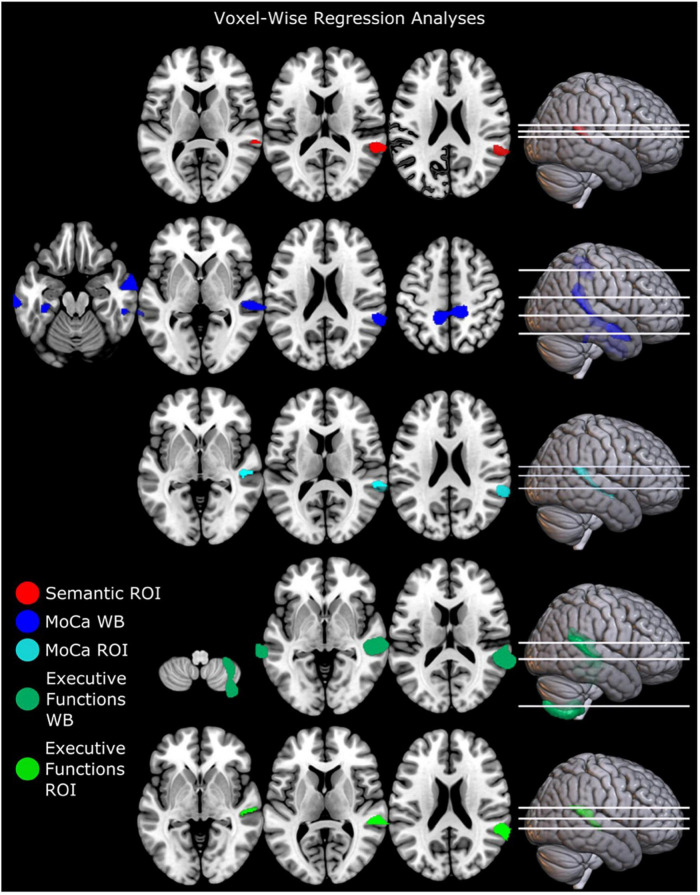
Regression analyses of brain structure and cognitive and linguistic factors in PD with mild and moderate dysarthria. Executive functions were calculated as a composite score (see Supplementary Material). Results are corrected for multiple comparisons with FWEc *p* < 0.05. ROI mask comparisons were only significant in the right hemisphere. Left is displayed left. Abbreviations: totDPD, PD with mild and moderate dysarthria; MoCA, Montreal Cognitive assessment; ROI, region of interest; Semantic, verbal fluency semantic score; WB, whole-brain comparison.

Structural brain alteration regressions were not significant for MDS-UPDRS total score, MDS-UPDRS motor part (III), QASD total score, self-rated dysarthria composite score, verbal fluency FAS score, AVQI, and voice sound level (see Supplementary Table 4 for summary statistics). Gray matter volume in totDPD correlated positively with MoCA in whole-brain and right ROI regression in the right superior temporal gyrus (Figure 1 top row and Supplementary Table 2). Lower executive function composite score correlated with decreased gray matter volume in totDPD in the whole-brain and right ROI mask in the right superior temporal gyrus. Decreased gray matter volume in totDPD in the right ROI mask correlated with lower verbal fluency semantic score. Since the groups differed in motor symptom severity (MDS-UPDRS III), we reanalyzed the statistically significant comparisons controlling for MDS-UPDRS III and obtained the same patterns.

To analyze if these results were driven by PD and not by the combination of PD with dysarthria, we performed regressions with the variables that were significantly correlated (semantic verbal fluency, MoCA, and executive functions composite score) in the whole-study group and in the noDPD group only. The analysis of the whole study group lead to the same patterns as described above. The noDPD regressions yielded no statistically significant results.

### Resting-State Alterations

#### Group Comparison

**FIGURE 2:**
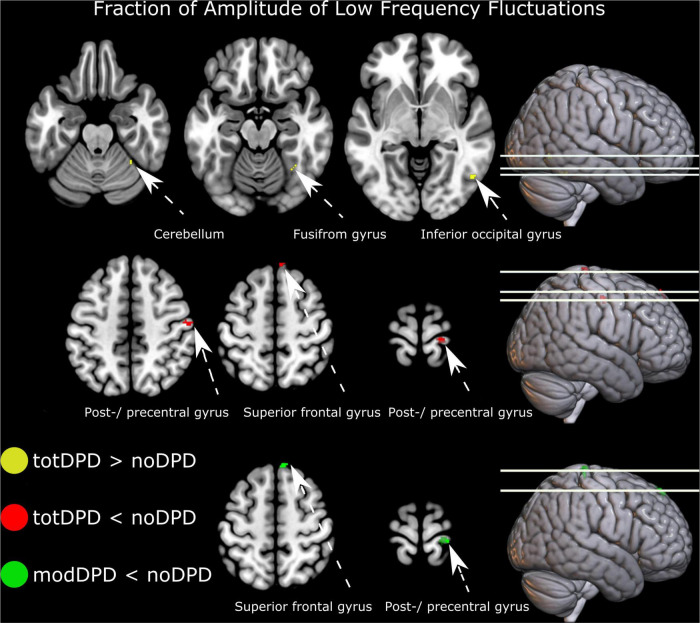
Resting-state alterations in participants with PD and dysarthria (DPD). From top to bottom: group comparison intensity of spontaneous brain activity (fraction of amplitude of low frequency fluctuations) in noDPD vs. totDPD and modDPD vs. noDPD. Results are corrected for multiple comparisons with FWEc *p* < 0.05. Left is displayed left. Abbreviations: mildDPD, PD with mild dysarthria; modDPD, PD with moderate dysarthria; noDPD, PD with no dysarthria; totDPD, PD with mild and moderate dysarthria.

Comparing noDPD with totDPD, zfALFF maps showed decreased regional spontaneous brain activity in the right postcentral gyrus and right superior frontal gyrus (Figure 2 top panel and Supplementary Table 2). Increased brain activity was found in the right inferior occipital gyrus and right cerebellum exterior/fusiform gyrus. When stratifying into dysarthria severity subgroups, noDPD vs. modDPD yielded the same pattern for decreases but not for increases in zfALFF measures (Figure 2 bottom panel and Supplementary Table 2). There were no statistically significant differences between noDPD and mildDPD. Further, ROI, ReHo and degree centrality analyses yielded no significant changes.

#### Regression Analyses

Regressions between intensity of spontaneous brain activity (zfALFF) and the following measures were not significant: MoCA, MDS-UPDRS III, executive functions composite score, verbal fluency semantic score, verbal fluency FAS score, AVQI, and voice sound level (see Table 2 for summary statistics).

## Discussion

The present study aimed at shedding light on mechanisms behind dysarthria in people with PD. There were differences in voice sound level, degree of dysphonia, and motor symptom severity (MDS-UPDRS III) between participants with different levels of severity of dysarthria. There were no statistically significant differences between PD with and without dysarthria in brain structure. However, lower resting-state brain activity related to PD with dysarthria was found in the right postcentral gyrus and superior frontal gyrus. Furthermore, higher brain activity in the right inferior occipital gyrus and cerebellum exterior/fusiform gyrus was related to PD with dysarthria. In addition, lower scores on measures of global cognition (MoCA), language (semantic verbal fluency), and executive function (composite score) were independently associated with decreased gray matter volume in the right superior and middle temporal cortices in PD with different levels of severity of dysarthria. Our study provides a valuable addition to the sparse literature on PD with dysarthria and related brain changes, and raises questions for further investigation.

### Brain Alterations in Parkinson’s Disease With Dysarthria

In our study, PD with dysarthria was characterized by focal decreased and increased resting-state brain activity when compared to PD without dysarthria. Decreased spontaneous brain activity intensity in moderate dysarthria was found in the right postcentral gyrus and superior frontal gyrus when compared to PD participants without dysarthria. A similar pattern was present when comparing the whole group of dysarthria to PD participants without dysarthria. These results need to be interpreted cautiously and should be seen as a tendency. Nevertheless, in the following, we discuss these tendencies in the light of the present evidence in the literature.

Comparable studies investigating speech symptoms and brain changes are sparse. Of note, the literature differs in defining speech impairment and measures of resting-state activity. In a seed-based rsMRI study, Manes and colleagues (Manes et al., 2018) investigated basal ganglia connectivity in participants with PD and speech impairment. The authors found lower connectivity between the left putamen and left superior temporal gyrus as well as higher connectivity between the internal globus pallidus and the left dorsal premotor/laryngeal motor cortex, left angular gyrus, and right angular gyrus when comparing PD with and without speech impairment. Data from the Parkinson’s Progression Markers Initiative database were explored including 42 PD participants without speech impairment and 35 PD participants with speech impairment based on MDS-UPDRS-III speech item scores. Of note, the groups marginally differed in motor symptom severity (MDS-UPDRS-III), but in correlation analyses of connectivity strength with motor severity, no significant relationship was found. Thus, it was possible to separate speech impairment defined by the MDS-UPDRS-III speech item from general motor severity. This is along the same line as our results; the groups noDPD, mildDPD, and modDPD, differed in motor severity, but we could not identify influences of MDS-UPDRS-III on brain alterations. On the other hand, in the study of Manes et al., speech impairment in PD seems to be related to dysconnectivity in the left-hemisphere basal ganglia, while in our study alterations were mainly found in the right hemisphere. Nevertheless, functions underlying speech and language are not just lateralized but processed in both hemispheres (Brabenec et al., 2017).

One of the most common symptoms in hypokinetic dysarthria is reduced voice sound level. This has been suggested to be a result of reduced motor drive caused by basal ganglia dysfunction (Fox et al., 2002). Moreover, changes in the perception of the loudness of the own voice have been shown in people with PD (Ho et al., 2000). It is common that people with PD and dysarthria perceive that they are screaming and are much too loud when asked to speak up, with difficulties scaling up and regulating the effort required to speak with normal intensity. Our cohort of participants with PD and dysarthria differed in voice sound level depending on dysarthria severity level. The mean voice sound level at a group level was 3.7 dB lower for modDPD and 2.1 dB lower for totDPD compared to noDPD, respectively. In line with our results, Fox and Ramig (1997) also performed controlled studio recordings of speech and voice in participants with PD, and a 2-4 dB lower voice sound level compared to controls was found. Arnold et al. (2014) hypothesized that the mechanism behind dysarthria could partially be a late consequence of pathological sensorimotor integration. They performed an fMRI study with a sentence reading task in 20 participants with PD (Hoehn and Yahr stage 1–2), without any sign of hypophonia or dysarthria and found changes in the interplay between the striatum and prefrontal cortices bilaterally as well as reduced activity in the auditory cortex in external auditory processing. Hence, speech networks may be altered in participants with PD even before they develop speech symptoms. Consequently, future studies investigating brain connectivity in PD and dysarthria should preferably include healthy controls for comparisons.

Our study identified functional, but no structural, differences between dysarthria severity groups. Yet, we found brain structure to be related to semantic verbal fluency, general cognition, and executive functions in our cohort. Important to note is that the functional changes were of focal sizes and need to be interpreted in a humble way. sMRI, as analyzed with voxel-based morphometry, reflects morphological changes, while rsMRI, as analyzed with zfALFF, symbolizes spontaneous brain activity (Ashburner and Friston, 2000; Chao-Gan and Yu-Feng, 2010). Our results can be interpreted using the framework of “molecular nexopathy.” It is hypothesized that “soft” changes–synaptic and related network activity alterations–give rise to subsequent “hard” and non-reversible changes–cell loss and thus brain structure alterations (Warren et al., 2013). In PD, as well as in other neurodegenerative diseases, it has been shown that functional changes antedate structural changes (Mosconi et al., 2006; Tang et al., 2013; Albrecht et al., 2019). Indeed, in a comprehensive meta-analysis of different PD subtypes, no consistent findings for sMRI could be obtained but specific functional changes as measured with positron emission tomography were found as a hallmark of PD (Albrecht et al., 2019). Further, in a quantitative neuroimaging meta-analysis of rsMRI comparing PD and healthy controls, common alterations in the bilateral inferior parietal lobule and the supramarginal gyrus were described (Tahmasian et al., 2017). Since our study cohort needed to be able to take part in several different assessments including MRI and an intervention 3 times a week for 10 weeks, they consisted of participants with mild-moderate PD, thus relatively early in the disease progression. Hence, the fact that functional but no structural alterations were found, is in line with the theory that functional changes may precede structural alterations in the disease progression.

### Superior Temporal Gyrus Involvement in Speech and Language

In this study, we found that structural changes in the superior temporal gyrus (STG) are related to measures of language (verbal fluency), global cognition (MoCA), and executive functions (composite score) in participants with PD and dysarthria. The STG has since long been regarded as an important cortical hub for speech and language (Wernicke, 1874; Geschwind, 1970). In recent years, studies have highlighted the role of STG in various aspects of auditory- and phonological processing as well as lexical representations. For example, the STG is a key brain region involved in auditory feedback, specifically by coding mismatches between expected and actual auditory signals (Parkinson et al., 2012; Flagmeier et al., 2014). Studies investigating the various contributions of STG to speech processing were reviewed by Yi et al. (2019) and the authors suggest that the STG may play a more substantial role in multiple aspects of speech perception than previously understood. Of note, resting-state connectivity studies have shown that the bilateral STG may also be involved in language functions, for example, lexical selection and semantic processing (Gertel et al., 2020). Other studies also found evidence that STG may be involved in speech impairment in PD. For example, New et al. (2015) used ROIs based on the same meta-analysis as in our study (Brown et al., 2009) in a rsMRI connectivity analysis of 56 participants with PD and 56 healthy controls. Interestingly they found decreased connectivity between the left thalamus, putamen, cortical motor areas, and right STG when comparing PD with controls. Noteworthy, more severe speech impairment was correlated with increased bilateral Rolandic operculum, left STG, and left cerebellum connectivity. In contrast, more severe speech impairment was also correlated to lower connectivity in the right STG, bilateral ventral premotor area, the right putamen, and left thalamus. The participants in our cohort presented structural changes in the STG related to speech symptoms, but no relating resting-state changes. Several methodological differences could contribute to the differing results between the study by New and colleagues and our study. First, the method of our study is different since we did not compare to healthy controls, but to other participants with PD. Thus, New and colleagues might have found general PD-related alterations. Second, the studies differ in their statistical analyses of resting-state: our study focused on voxel-wise zfALFF while the study by New and collegues assessed between-region connectivity. Third, in the study by New and colleagues measures of speech impairment were derived from general PD assessment, e.g., item 5 from MDS-UPDRS II (rating of speech impairment within the context of activities of daily living). In contrast, we utilized perceptual ratings of expert listeners in the assessment of speech impairment which we argue is a more valid method of assessing dysarthria in PD.

Of note, we found no differences between groups with different levels of severity of dysarthria in the measures of semantic verbal fluency, the composite score for executive functions, and MoCA. Therefore, we found no evidence in our cohort that lower scores on these tests are associated with dysarthria in PD. The structural changes in the STG in relation to lower scores were found in PD participants with mild to moderate dysarthria, but not in PD participants without dysarthria. However, the group of participants without dysarthria was small (*n* = 19). Consequently, the statistical power may have been too low to detect structural changes in relation to language, cognition, and executive functions in this group. Controlling for motor symptom severity (MDS-UPDRS III) yielded the same patterns of structural changes in the STG, which suggests that the results may not solely be driven by general disease severity. However, we recommend hypothesis-driven studies to further investigate the role of STG in dysarthria in people with PD.

### Limitations

We identified changes in PD participants with dysarthria and the analyses were performed to the best of our knowledge, still, limitations should be acknowledged. One of the inclusion criteria for the EXPANd RCT was a MoCA score ≥ 21. Hence, there is a ceiling effect that should be considered when interpreting the analyses in this study involving the MoCA score. Also due to the inclusion criteria for the EXPANd RCT, the participants did not have severe dysarthria. Thus, the results from this study may not necessarily apply to all individuals with PD and dysarthria. Furthermore, for comparisons, a cohort of age-matched healthy control participants performing the same tests and measures would have been advantageous. We acknowledge that the sample size is small for an MRI study and results should be interpreted with caution. Our unequal group sizes may, unfortunately, lead to less power to detect differences since power relies on the smallest group. Especially, the rsMRI results of our study should be interpreted with caution since these were more exploratory. Statistically significant changes of focal size were found for zALFF but not for other measures (ReHo and degree centrality). We further acknowledge that all participants of the EXPANd trial were assessed in the ON stage of dopaminergic medication. Thus, medication could have influenced our measurement, which could have especially influenced speech impairment and motor symptom severity (MDS-UPDRS). However, the EXPANd trial aimed to investigate the participants with PD in a state that they are in most of the day. Since the cohort presented with a relatively mild disease the participants were mostly in an ON state. Consequently, we argue that assessments in the ON state can be considered ecologically valid.

In this manuscript, we used perceptual listener ratings for assessing dysarthria, attempting to shed light on symptoms of mild dysarthria in this cohort of participants with mild-moderate PD. The parameters rated in the participants’ speech samples were articulation, prosody, voice function, and overall impression. We validated the perceptual rating of the parameter voice function using multiple linear regression analysis with measures of voice sound level and dysphonia (AVQI). Unfortunately, in this study, we have no objective measures of the rated parameters articulation and prosody. Hence, there is a need for further validation of this method for the assessment of dysarthria. Furthermore, we acknowledge that our study can only draw conclusions about the combination of PD and dysarthria. Further investigations might be necessary to disentangle the general disease profile from speech impairment. Namely, the significant correlations of measures of language and cognition with gray matter volume may be present in the entire PD population and not only related to dysarthria.

## Conclusion

The results of this study show that different levels of severity of dysarthria in PD are related to differences in voice sound level, dysphonia, and motor symptom severity. Furthermore, the results suggest that dysarthria in PD is associated with focal brain changes in resting-state but not gray matter volume changes. Still, clinical measurements of language and cognition such as semantic verbal fluency, global cognition, and executive functions were shown to be associated with lower gray matter volume in the temporal lobe in participants with PD and mild-moderate dysarthria. We highlight the need for new approaches of assessing dysarthria in mild-moderate PD to pave the way for better comparability between studies. Future studies should focus on disentangling dysarthria from general disease severity in relation to language and cognition in PD.

## Data Availability Statement

Data were collected within the framework of the EXPANd RCT, and the study protocol is published (Franzén et al., 2019). The statistical analysis plan of the current study was preregistered at aspredicted.org and is available on the EXPANd RCT OSF page together with the statistical analysis scripts (osf.io/s952g). The data generated during the current study are not publicly available due to Swedish and EU personal data legislation. Upon reasonable request, data are available from the corresponding author. Sharing of the data will be regulated via a data transfer and user agreement with the recipient.

## Ethics Statement

The studies involving human participants were reviewed and approved by Regional Ethical Review Board in Stockholm. The participants provided their written informed consent to participate in this study.

## Author Contributions

HS: data collection, study design, data analysis of behavioral data, and manuscript writing. ES and EF: data collection, study design, funding, and contributions to manuscript. FA: study design, data analysis of neuroimaging and behavioral data, and manuscript writing. All authors contributed to the article and approved the submitted version.

## Conflict of Interest

The authors declare that the research was conducted in the absence of any commercial or financial relationships that could be construed as a potential conflict of interest.

## Publisher’s Note

All claims expressed in this article are solely those of the authors and do not necessarily represent those of their affiliated organizations, or those of the publisher, the editors and the reviewers. Any product that may be evaluated in this article, or claim that may be made by its manufacturer, is not guaranteed or endorsed by the publisher.

## Footnotes

1
osf.io/s952g

## Supplementary Material

Supplementary Methods Additional details on rsMRI processing.

Supplementary Results R output for the intra-rater reliability and results for two other rsMRI measures.
