# Supplementary material for: Characterization of Mild and Moderate Dysarthria in Parkinson’s Disease: Behavioral Measures and Neural Correlates
Source: Front Aging Neurosci. 2022 May 16;14:870998. doi: 10.3389/fnagi.2022.870998 (PMC9148995; doi:10.3389/fnagi.2022.870998)
Supplement: Supplementary Methods — Additional details on rsMRI processing. [file Data_Sheet_1.docx]

Table of Contents

[SUPPLEMENTAL MATERIAL to “Characterization of mild and moderate dysarthria in Parkinson’s disease: behavioral measures and neural correlates” 1](#_Toc98431659)

[METHODS 1](#_Toc98431660)

[Resting-state MRI 1](#_Toc98431661)

[Degree Centrality 1](#_Toc98431662)

[Regional homogeneity (ReHo): 1](#_Toc98431663)

[RESULTS 1](#_Toc98431664)

[Intra-rater reliability R output 1](#_Toc98431665)

[Resting-state MRI 2](#_Toc98431666)

[Supplemental Tables 2](#_Toc98431667)

[**Supplemental Table 1** Results from the validity and reliability analysis. 2](#_Toc98431668)

[**Supplemental Table 2** Description of the presence of dyskinesia stratified into the groups with different severity levels of dysarthria, assessed with the MDS-UPDRS item 3.18A. 3](#_Toc98431669)

[**Supplemental Table 3** Description of the proportion of participants with mild cognitive impairment (MCI) in the total sample as well as stratified into the groups with different severity levels of dysarthria. 3](#_Toc98431670)

[**Supplemental Table 4** Resulting peak coordinates of group comparison and regression analyses in sMRI and rsMRI. 3](#_Toc98431671)

# METHODS

## Resting-state MRI

Degree Centrality*:* measures the temporal correlation between a voxel and all other voxels of the brain. RsMRI data was nuisance regressed with a linear model which was constructed with simultaneous bandpass filtering (0.01 - 0.1Hz). A matrix of Pearson’s correlation coefficients was computed on the unsmoothed data with a threshold for the Pearson’s correlation coefficient of r > 0.25. We analyzed the z-standardized, binarized degree centrality values of the whole-brain. Afterwards images were smoothed with FWHM=8mm.

Regional homogeneity (ReHo): a voxel-wise computation of homogeneity between a voxel and its neighboring voxels. The similarity of the time series of a voxel is compared to the 27 surrounding voxels. RsMRI data was nuisance regressed with a linear model which was constructed with simultaneous bandpass filtering (0.01 - 0.1Hz). We analyzed the z-standardized ReHo maps which were smoothed thereafter with FWHM=8mm.

# RESULTS

## Intra-rater reliability R output

[1] "The measurement scale is nominal."

[1] "###### Fleiss' K ######"

[1] "The observed agreement in all complete cases is 59.1%."

[1] "N (number of subjects without missing values) = 22"

[1] "n (number of ratings) = 2"

[1] "k (number of categories) = 8"

[1] "Point estimator of Fleiss' K = 0.4864"

[1] "Asymptotic two-sided 95% confidence interval for Fleiss' K: 0.2967 ; 0.6761"

[1] "Two-sided 95% Bootstrap confidence interval for Fleiss' K: 0.2022 ; 0.7277"

[1] "###### Krippendorff's alpha ######"

[1] "The observed agreement in all cases with at least two ratings is 59.1%."

[1] "N (number of subjects with two or more ratings) = 22"

[1] "n (number of ratings) = 2"

[1] "k (number of categories) = 8"

[1] "Point estimator of Krippendorff's alpha = 0.4981"

[1] "Two-sided 95% Bootstrap confidence interval for Krippendorff's alpha: 0.2224 ; 0.7306"

## Resting-state MRI

There were no significant group differences in the ReHo and degree centrality maps between the groups. Regarding regression analyses, we correlated only zfALFF maps with behavioral variables since we did not see any group differences in ReHo or degree centrality maps.

# Supplemental Tables

## **Supplemental Table 1** Results from the validity and reliability analysis.

|  | Adj R2 | B | SE B | *std*  b | *p* | *95% CI*  *L.L.* | *95% CI U.L.* |
| --- | --- | --- | --- | --- | --- | --- | --- |
| Variable | 0.116 |  |  |  |  |  |  |
| *Constant* |  | 4.286 | 1.371 |  | 0.002** | 1.556 | 7.015 |
| AVQI |  | 0.106 | 0.081 | 0.142 | 0.195 | -0.055 | 0.266 |
| Voice sound level |  | -0.051 | 0.018 | -0.311 | 0.005** | -0.087 | -0.016 |

## **Supplemental Table 2** Description of the presence of dyskinesia stratified into the groups with different severity levels of dysarthria, assessed with the MDS-UPDRS item 3.18A.

|  | **mild dysarthria (N=44)** | **moderate**  **dysarthria (N=20)** | **no dysarthria**  **(N=19)** | **Total (N=83)** |
| --- | --- | --- | --- | --- |
| **Missing values** | 2 | 2 | 1 | 5 |
| **No (0)** | 37 (88.1%) | 16 (88.9%) | 17 (94.4%) | 70 (89.7%) |
| **Yes (1)** | 5 (11.9%) | 2 (11.1%) | 1 (5.6%) | 8 (10.3%) |

## **Supplemental Table 3** Description of the proportion of participants with mild cognitive impairment (MCI) in the total sample as well as stratified into the groups with different severity levels of dysarthria.

|  | **mild dysarthria (N=44)** | **moderate dysarthria (N=20)** | **no dysarthria (N=19)** | **Total (N=83)** |
| --- | --- | --- | --- | --- |
| **Non-MCI** | 32 (72.7%) | 13 (65.0%) | 14 (73.7%) | 59 (71.1%) |
| **MCI** | 12 (27.3%) | 7 (35.0%) | 5 (26.3%) | 24 (28.9%) |

## **Supplemental Table 4** Resulting peak coordinates of group comparison and regression analyses in sMRI and rsMRI.

| **Cluster p-value (FWEc)** | **Voxel number** | **T** | **Equiv Z** | **peak p-value (unc)** | **x,y,z {mm}** |
| --- | --- | --- | --- | --- | --- |
| **fALFF totDPD < noDPD** |  |  |  |  |  |
| 0.029 | 77 | 4.99 | 4.60 | <0.001 | 18 -30 76 |
| 0.045 | 70 | 4.69 | 4.36 | <0.001 | 54 -14 48 |
| 0.035 | 74 | 4.30 | 4.04 | <0.001 | 10 42 56 |
|  |  | 3.79 | 3.61 | <0.001 | 8 46 48 |
| **fALFF totDPD > noDPD** |  |  |  |  |  |
| 0.027 | 78 | 4.57 | 4.26 | <0.001 | 48 -64 -2 |
|  |  | 3.91 | 3.70 | <0.001 | 42 -74 -10 |
| 0.024 | 80 | 4.23 | 3.98 | <0.001 | 32 -50 -22 |
|  |  | 4.16 | 3.92 | <0.001 | 36 -54 -16 |
|  |  | 3.58 | 3.42 | <0.001 | 26 -58 -18 |
| **fALFF modDPD < noDPD** |  |  |  |  |  |
| 0.002 | 112 | 5.36 | 4.43 | <0.001 | 32 -30 68 |
|  |  | 4.98 | 4.20 | <0.001 | 24 -32 76 |
| 0.001 | 119 | 5.22 | 4.35 | <0.001 | 8 42 54 |
|  |  | 4.34 | 3.78 | <0.001 | 0 32 56 |
| **Positive Regression totDPD Semantic ROI** |  |  |  |  |  |
| 0.030 | 1065 | 4.82 | 4.41 | <0.001 | 58 -42 15 |
| **Positive Regression totDPD MoCA WB** |  |  |  |  |  |
| <0.001 | 5653 | 5.42 | 4.87 | <0.001 | 60 -39 14 |
|  |  | 5.17 | 4.68 | <0.001 | 66 -2 -15 |
|  |  | 3.98 | 3.73 | <0.001 | 62 10 -24 |
| 0.007 | 3130 | 4.47 | 4.13 | <0.001 | -10 -46 54 |
|  |  | 4.38 | 4.07 | <0.001 | -16 -45 46 |
|  |  | 4.27 | 3.97 | <0.001 | 20 -33 51 |
| 0.024 | 2194 | 4.33 | 4.02 | <0.001 | -32 0 -33 |
|  |  | 4.04 | 3.78 | <0.001 | -34 -33 -21 |
| 0.022 | 2245 | 4.12 | 3.85 | <0.001 | -64 -18 -10 |
|  |  | 4.02 | 3.77 | <0.001 | -70 -24 -8 |
|  |  | 3.73 | 3.52 | <0.001 | -54 -6 -27 |
| **Positive Regression totDPD MoCA ROI** |  |  |  |  |  |
| 0.007 | 1934 | 5.42 | 4.87 | <0.001 | 60 -39 14 |
|  |  | 5.14 | 4.66 | <0.001 | 68 -3 -14 |
|  |  | 4.25 | 3.96 | <0.001 | 68 -32 2 |
| **Positive Regression totDPD Executive functions WB** |  |  |  |  |  |
| 0.002 | 4233 | 5.60 | 5.01 | <0.001 | 58 -40 14 |
|  |  | 4.47 | 4.14 | <0.001 | 64 -30 0 |
| 0.028 | 2177 | 4.73 | 4.34 | <0.001 | -69 -32 -24 |
|  |  | 4.17 | 3.89 | <0.001 | -72 -30 -4 |
|  |  | 3.81 | 3.59 | <0.001 | -69 -48 -15 |
| 0.029 | 2169 | 3.96 | 3.72 | <0.001 | 38 -54 -63 |
|  |  | 3.59 | 3.40 | <0.001 | 36 -76 -57 |
| **Positive Regression totDPD Executive functions ROI** |  |  |  |  |  |
| 0.004 | 2326 | 5.60 | 5.01 | <0.001 | 58 -40 14 |
|  |  | 4.47 | 4.14 | <0.001 | 63 -28 0 |
| Abbreviations: Executive functions Composite score of measuring executive functions, mildDPD Parkinson’s disease with mild dysarthria, modDPD Parkinson’s disease with moderate dysarthria, noDPD Parkinson’s disease without dysarthria, ROI region of interest, totDPD Parkinson’s disease with mild to moderate dysarthria, WB whole-brain. | | | | | |
